# Impairment of the autophagy-lysosomal pathway and activation of pyroptosis in macular corneal dystrophy

**DOI:** 10.1038/s41420-020-00320-z

**Published:** 2020-09-12

**Authors:** Tao Zheng, Chuchu Zhao, Baowen Zhao, Hanruo Liu, Shijian Wang, Liyuan Wang, Ping Liu

**Affiliations:** 1 Department of Ophthalmology, The First Affiliated Hospital of Harbin Medical University, Harbin, 150001 China; 2 The Beijing Institute of Ophthalmology, Beijing Tongren Hospital, Capital Medical University, Beijing, 100730 China

**Keywords:** Inflammasome, Gene expression, Metabolic disorders, Disease genetics

## Abstract

Macular corneal dystrophy (MCD) is ascribed to mutations in the carbohydrate sulfotransferase (*CHST6*) gene affecting keratan sulfate (KS) hydrophilicity and causing non-sulfated KS to precipitate in keratocytes and the corneal stroma. We investigated roles for inflammatory responses in MCD pathogenesis by examining the lysosomal-autophagy pathway and activation of pyroptosis in MCD keratocytes. Normal and lesioned keratocytes were obtained from MCD patients undergoing corneal transplantation. The keratocytes were subjected to gene sequencing, RT-PCR, western blotting, transmission electron microscopy, histological staining, induction and inhibition assays of autophagy and pyroptosis, CCK-8 and LysoTracker Green DND-26 labeling, and flow cytometry. A novel homozygous MCD mutation was identified in a family from Northeast China; the mutation was distinguished by cytoplasmic vacuolation, cell membrane disruption, electron dense deposits, and deposition of a band of Periodic acid-Schiff and Alcian blue-positive material in the keratocytes and stroma layer. KS protein levels were decreased, expression of p62 and LC3-II proteins was enhanced, cathepsin D expression was declined and the LysoTracker Green DND-26 signal was dramatically reduced in MCD keratocytes. Bafilomycin-A1 treatment significantly increased caspase-1 and Pro-IL-1β expression in normal and MCD keratocytes. Nod-like receptors pyrins-3 (NLRP3), caspase-1, Pro-IL-1β, and IL-1β levels were pronouncedly elevated in cells exposed to H_2_O_2_. Ac-YVAD-CMK treatment reversed this expression in normal and MCD keratocytes. Suppression of the autophagic degradation of non-sulfated KS by impaired autophagic flux in MCD keratocytes triggers pyroptosis. Amelioration of impaired autophagy and restraint of pyroptosis may, therefore, have therapeutic efficacy in the treatment of MCD.

## Introduction

Macular corneal dystrophy (MCD, OMIM217800), also known as Groenouw II type corneal dystrophy, is a rare autosomal recessive-inherited condition characterized by progressive loss of vision, photophobia, and discomfort on the ocular surface. The symptom of a diffuse, foggy corneal stroma progresses from a focal, patchy, white opacity with unclear boundaries to keratoleukoma in both eyes and finally cornea thinning, as determined by ocular slit lamp examination^1^. MCD has a high prevalence in India, Saudi Arabia, and Iceland due to increased rates of mutation in the *CHST6* gene, and this prevalence can largely be ascribed to consanguinity^2^. The most effective short-term treatment for recovery of sight acuity in MCD is keratoplasty and deep anterior lamellar corneal transplantation^3^. However, recurrence after keratoplasty and complications such as graft rejection and endothelial cell loss following transplantation have been documented^4^. Research on gene and drug therapies for MCD is, therefore, urgently required.

The pathogenic gene of MCD is *CHST6*, which encodes corneal N-acetylglucosamine-6-sulfphotransferase (GlcNAc6ST) and is located on chromosome 16 (16q22) (ref. ^5^). Sulfated KS, formed by the action of GlcNAc6ST, is transported to the extracellular matrix (ECM), where it participates in the formation of some components of the corneal stroma and serves as a critical corneal adhesive^6,7^. Sulfated KS is abundantly present in the corneal stromal layer, the pro-elastic layer, and the post-elastic layer; however, expression of sulfated KS is low in the epithelium, endothelium, and keratocyte in normal corneal tissues^8^. Mutation of the *CHST6* gene results in accumulation of large amounts of non-sulfated KS (usually absent in the normal cornea) and its deposition in the keratocytes and in the ECM of the corneal stroma, leading to keratocytes injury and corneal tissue opacity^5,9^.

Accumulation of non-sulfated KS due to the *CHST6* gene mutation may also affect the process of pyroptosis, also known as inflammatory necrosis. Pyroptosis is a kind of programmed cell death characterized by the continuous expansion of cells until the rupture of cell membranes leads to the release of intracellular contents and activation of a strong inflammatory response^10^. Pyroptosis is an important innate immune response triggered by various pathological stimuli, including microbial infections, cardiovascular diseases, central nervous system diseases, and malignant tumors^11–14^. The process begins with discernment of pathogen-related molecular patterns or damage-related molecular patterns (DAMPs) by Toll-like receptors as a priming or initiating signal. This triggers signaling by nuclear factor kappa B, which in turn upregulates transcription of inflammasome-related components, including inactive NLRP3, pro-IL-1β, and pro-IL-18 (refs. ^15–18^). Gasdermin-D (GSDMD), a newly identified executioner of pyroptotic cell death, is cleaved by activated caspase-1 catalyzed by the NLRP3 inflammasome. Activated caspase-1 also cleaves pro-IL-1β and pro-IL-18 into mature IL-1β, and IL-18, which are released through GSDMD pores^19^.

Depositions of non-sulfated KS in the keratocytes eventually need to be degraded. A fine balance is required between the synthesis and degradation of macromolecules to maintain intracellular homeostasis. Turnover of intracellular components is subtly mediated and involves their delivery to lysosomes rich in hydrolases to form an autolysosome^20^. Lysosomes, which are cellular organelles, play a fatal role in the degradation of macromolecules. To exert this degradative function, these organelles contain a variety of hydrolases in which cathepsins, a group of proteases enclosed in lysosomes, execute integral roles in producing lysosomal catabolites. Cathepsin D (CTSD) deficiency leads to the abnormal degradation of biological macromolecules in the body and their storage in lysosomes^21^. Currently, several mutation types of *CHST6*-associated MCD have been discovered in Northeast China^22^. The present study reports the first homozygous MCD mutant in Northeast China. Some reports of *CHST6* variants resulting in MCD have been published, but the pathogenesis of MCD remains underexplored^2^. However, its pathological characteristics hint at the possible involvement of a strong inflammatory response. Non-sulfated KS is a DAMP molecule, so it may activate the pyroptosis pathway, leading to keratocyte membranes rupture and strong inflammation. Keratocytes show changes in their appearance following stimulation with pyroptosis inducers and inhibitors. In addition, a distinct lysosome dysfunction is observed in MCD keratocytes, which again may reflect a disturbance due to pyroptosis. The aim of the present study was to investigate the mechanisms underlying the progression of MCD to further elucidate the pathogenesis of MCD and to provide a reliable basis for future gene therapy and drug treatment of MCD.

## Results

### Reduced synthesis of sulfated KS and abnormal lysosome function in MCD keratocytes

**Fig. 1:**
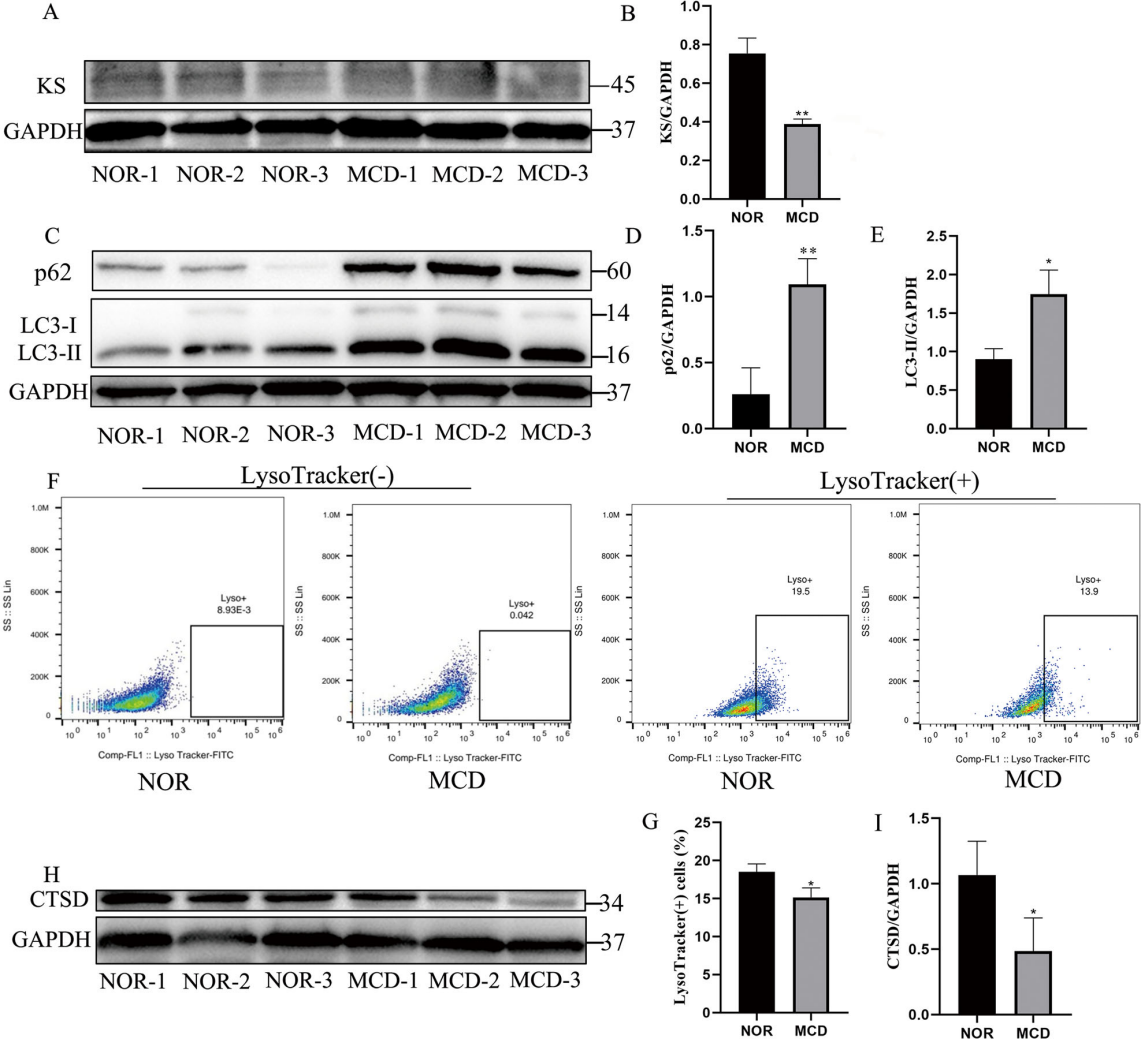
Sulfated keratan sulfate synthesis and lysosome function in normal (NOR) and MCD keratocytes. We analyzed three cell lines of various MCD patients (MCD-1, MCD-2, and MCD-3) and three cell lines of various NOR controls (NOR-1, NOR-2, and NOR-3). Western blot analyses of expression of sulfated KS in NOR and MCD keratocytes (**a**). **p < 0.01 versus NOR keratocytes; Student’s *t* test; *n* = 3 derived from each of three lines (**b**). Expression of p62 and LC3-II without any H_2_O_2_ stimulation in NOR and MCD keratocytes (**c**). *p < 0.05, **p < 0.01 versus NOR keratocytes; Student’s *t* test; *n* = 3 derived from each of three lines (**d**, **e**). FACS analysis of lysosome function using LysoTracker Green. ^#^*P* < 0.05, NOR vs. MCD; Student’s *t* test; *n* = 3 derived from each of three lines (**f**, **g**). CTSD protein level was measured using western blot in NOR and MCD keratocytes (**h**). *p < 0.05 versus NOR keratocytes; Student’s *t* test; *n* = 3 derived from each of three lines (**i**). Error bars indicate SD.

In MCD type II (MCD II), the cornea and serum show antigenic KS responses^23^ but at levels lower than normal^24,25^. The measurements of sulfated KS in the present study indicated a higher expression of sulfated KS protein in normal keratocytes than in MCD keratocytes (Fig. 1a, b). The TEM images indicated accumulations of deposits in the stromal ECM as well as in the endoplasmic reticulum (ER) of the MCD keratocytes^26^, indirectly confirming that a large number of unsulfated KS was deposited in the MCD keratocytes.

The non-sulfated KS, as an anomalous compound in MCD keratocytes, was also probably metabolized by autophagy. We detected expression of the autophagy biomarker, LC3. C-terminal processing of LC3 produces LC3-I, which is modified to LC3-II with the initiation of autophagosome formation. We also detected p62 expression, which indicates autophagic flux^27^. The expression levels of p62 and LC3-II proteins were higher in MCD keratocytes than in normal keratocytes, hinting at an acceleration of autophagy and impairment of autophagic flux in MCD keratocytes (Fig. 1c–e).

Optimum lysosome function is best achieved at an acidic pH. LysoTracker green is a lysosomophilic dye that allows monitoring of the pH sensitivity index of lysosomal function^28^. MCD keratocytes showed a loss of the LysoTracker signal when compared with similar observations in normal keratocytes (Fig. 1f, g), indicating a change in the lysosomal pH in MCD keratocytes. Lysosomal cathepsin activity is commonly used as an indicator of lysosomal function, and the CTSD, an aspartyl protease, plays a critical role in the degradation of macromolecules within autolysosomes^29^. The expression of CTSD at the protein level was higher in normal keratocytes than in MCD keratocytes (Fig. 1h, i), suggesting that autophagosomes accumulated because of the inhibition of autophagic degradation, e.g., because of a blockage of autophagosome-lysosome fusion or mitigation of lysosome content digestion due to decreased levels of CTSD^30^. The changes in lysosomal pH has been postulated to indicate that CTSD is a first-order aspartic acid protease with a particularly acidic optimum pH and pH-dependent maturity^31^, which would further account for the decreased CTSD in MCD keratocytes.

### Bafilomycin-A1 effects on autophagy and pyroptosis in normal and MCD keratocytes

**Fig. 2:**
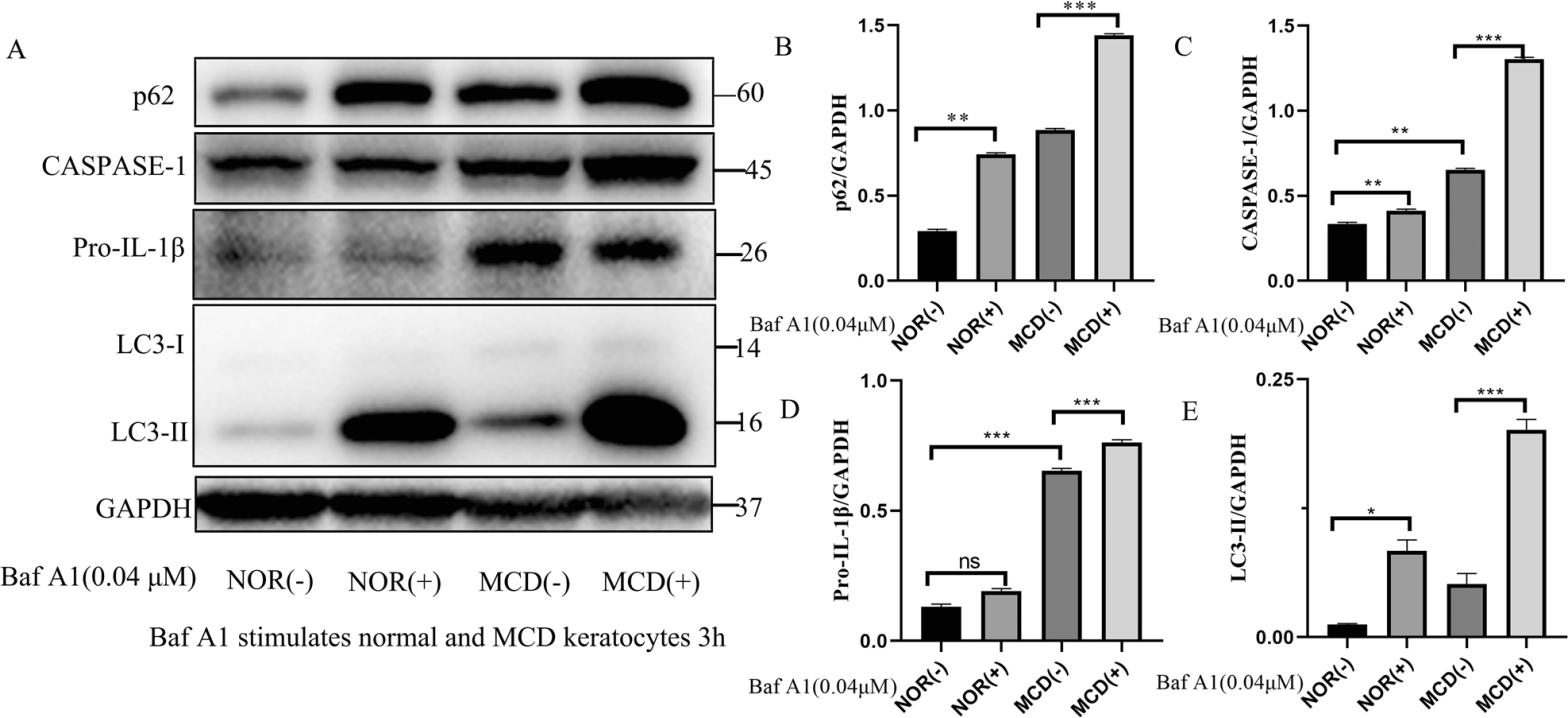
Trafficking defects and pyroptosis in NOR and MCD keratocytes. Expression of p62, LC3-II, caspase-1, and Pro-IL-1β in the presence or absence of bafilomycin-A1 (Baf A1, 0.04 μM) in NOR and MCD keratocytes (**a**). *p < 0.05, **p < 0.01, ***p < 0.001, ns = no significance versus NOR(−) and MCD(−); *n* = 3 for each group; One-way ANOVA (**b**–**e**). Error bars indicate SD.

Bafilomycin-A1, an inhibitor of the vacuolar H^+^-ATPase^32^, boosted p62 and LC3-II protein levels in normal and MCD keratocytes, but the increase was greater in the MCD keratocytes. This finding suggested that autophagy was facilitated and autophagic flux was blocked independent of the fusion of the autophagosomes with lysosomes in the MCD keratocytes (Fig. 2a, b, e). Pyroptosis differs from other types of cell death programs as it relies on caspase-1-mediated pathways. Bafilomycin-A1 significantly enhanced caspase-1 and Pro-IL-1β protein levels in MCD keratocytes but not in normal keratocytes. The levels of the pyroptosis-related proteins caspase-1 and Pro-IL-1β were still dramatically increased in MCD keratocytes even in the absence of bafilomycin-A1, indicating a role for pyroptosis in MCD (Fig. 2a, c, d). Taken together, these results indicated a linkage between autophagy and pyroptosis in MCD, whereby impairment of autophagy accelerated pyroptosis. This suggested that MCD keratocytes with impaired autophagy may have an increased susceptibility to cell death and that non-sulfated KS rarely exists in normal keratocytes.

#### Morphological hallmarks of MCD keratocytes

**Fig. 3:**
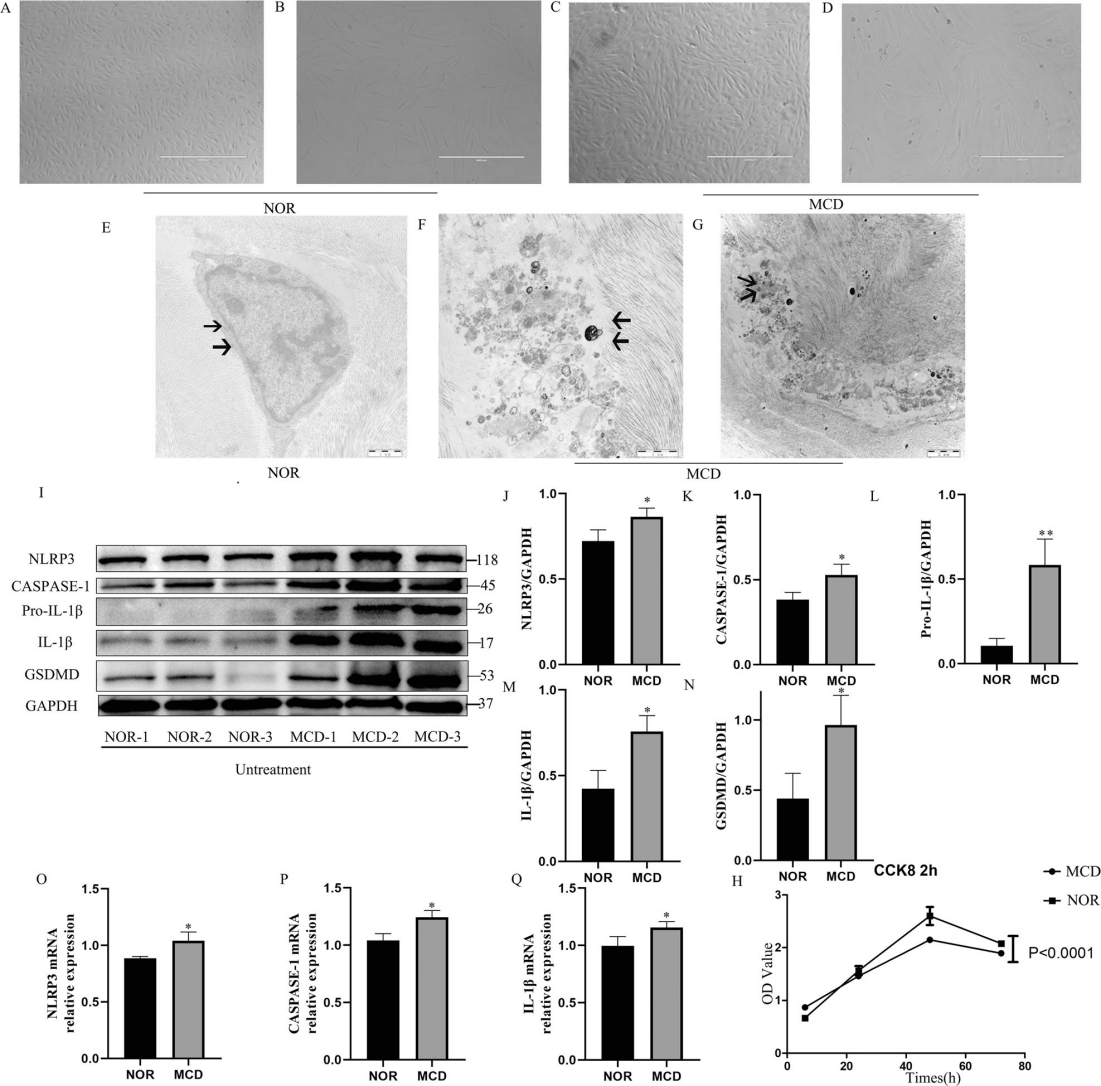
Cell morphology, ultrastructure, and the rate of cell growth of MCD keratocytes observed by phase-contrast microscopy, TEM, and CCK8 assays. Without any intervention, the expression of pyroptosis-related protein and mRNA was detected in NOR and MCD keratocytes by western blotting and RT-PCR. The morphology of the NOR and MCD keratocytes changes (**a**–**d**). As indicated by the arrow, a large number of electron-dense deposits and incomplete cell membranes (indicated by the arrow) were observed in MCD keratocytes, whereas normal keratocytes had intact cell membranes (indicated by the arrow) (**e**–**g**). Cell proliferation assays between the MCD and NOR keratocytes. *P* < 0.0001, MCD vs. NOR; Student’s *t* test; *n* = 3 in each group (**h**). Western blotting confirmed the differential expression of pyroptosis-related proteins (NLRP3, caspase-1, Pro-IL-1β, IL-1β, and GSDMD) in MCD keratocytes versus NOR keratocytes (*p < 0.05, **p < 0.01, MCD vs. NOR; Student’s *t* test; *n* = 3 in each group) (**i**–**n**). RT-PCR showed the expression of pyroptosis-related mRNA for NLRP3, caspase-1, and IL-1β in MCD keratocytes versus NOR keratocytes (*p < 0.05, MCD vs. NOR; Student’s *t* test; *n* = 3 in each group) (**o**–**q**). Error bars indicate SD. (Scale bars: 1 μm in **e**, **f**; 2 μm in **g**).

Phase-contrast microscopy revealed that MCD keratocytes were enlarged, longer, and had an irregular arrangement when compared with normal keratocytes (Fig. 3a–d). The pathological changes may occur in MCD keratocytes at the subcellular (organelle) level. The TEM images revealed ultrastructural degenerative changes, including vacuole formation, incomplete cell membrane, and electron dense deposits, in MCD keratocytes that were almost absent from normal keratocytes (Fig. 3e–g). The phenotype of the MCD keratocytes was characterized by evaluating the growth rate and cell death rate of the MCD keratocytes. The rate of cell growth was significantly lower in the MCD than in the normal keratocytes (Fig. 3h). The morphological changes consisted with recent descriptions of pyroptotic cells, which are characterized by swelling, fragmentation of the cell membranes, and progression to cell rupture that causes an inflammatory reaction.

#### Pyroptosis markers were increased in cultured keratocytes from MCD patients

Caspase-1 is activated via the inflammatory initiation factor NLRP3 that drives GSDMD pore formation on the membrane and ultimately results in programmed execution of cell death^33^. Measurement of pyroptosis-representative proteins by western blotting revealed significantly higher levels of NLRP3, caspase-1, Pro-IL-1β, IL-1β, and GSDMD in MCD keratocytes than in normal keratocytes (Fig. 3i–n). RT-PCR analyses showed upregulated expression of pyroptosis marker mRNAs in MCD keratocytes (Fig. 3o–q), confirming a possible involvement of pyroptosis in the *CHST6* mutation associated with MCD.

### A NLRP3 inducer (H_2_O_2_) exacerbated cell pyroptosis in both normal and MCD keratocytes

**Fig. 4:**
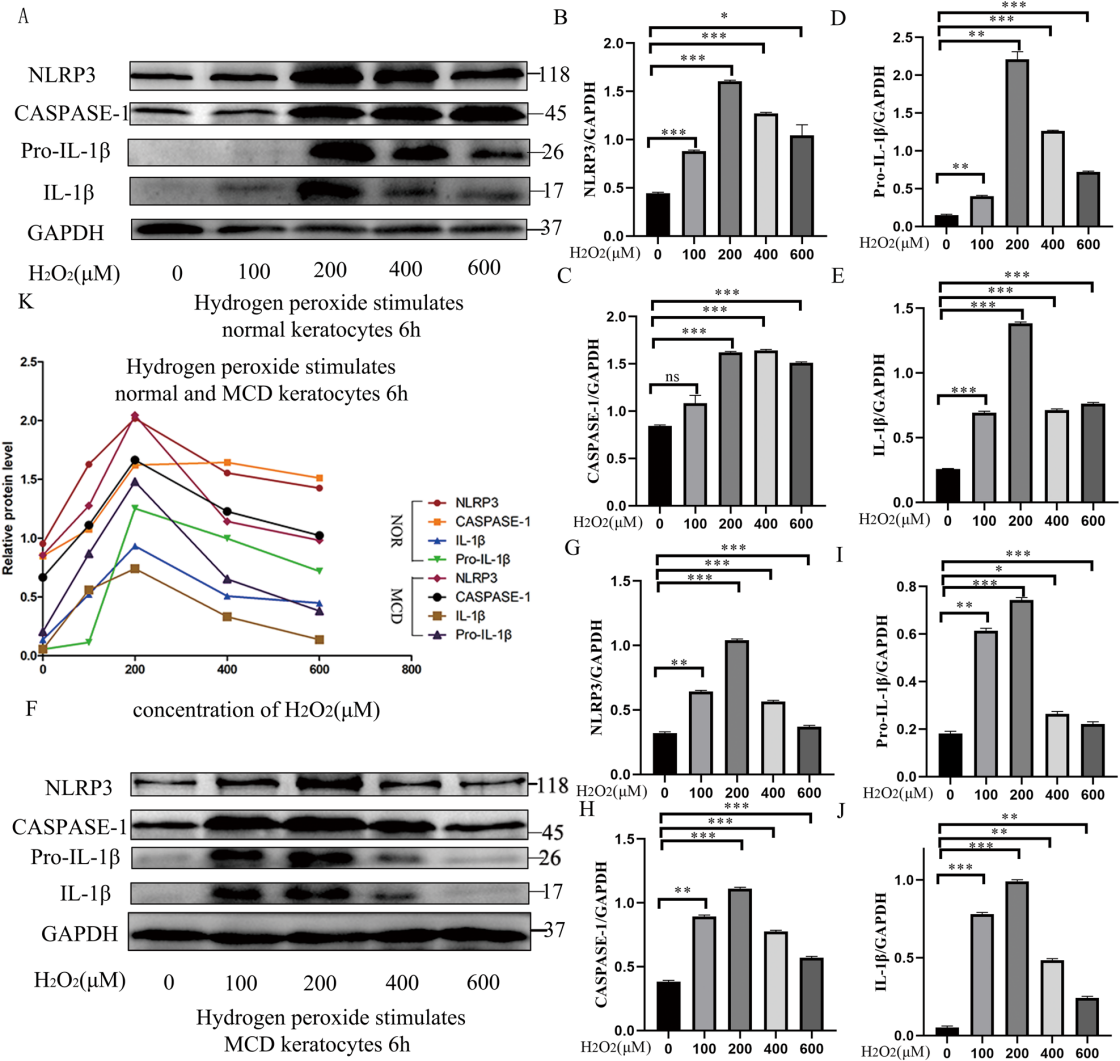
Assessment of the levels of pyroptosis in NOR and MCD keratocytes following a 6 h induction with different concentrations of H_2_O_2_. The changes in expression of pyroptosis-related proteins (NLRP3, caspase-1, Pro-IL-1β, and IL-1β) in the H_2_O_2_-induced normal keratocytes were detected by western blotting (**a**). *p < 0.05, **p < 0.01, ***p < 0.001, ns = no significance versus uninduced normal group (0 μM H_2_O_2_); *n* = 3 for each group; One-way ANOVA (**b**–**e**). In the case of MCD keratocytes induced with H_2_O_2_, the changes in expression of pyroptosis-related proteins (NLRP3, caspase-1, Pro-IL-1β, and IL-1β, were observed by western blotting (**f**). *p < 0.05, **p < 0.01, ***p < 0.001 versus uninduced MCD group (0 μM H_2_O_2_); *n* = 3 for each group; One-way ANOVA (**g**–**j**). A summary line chart indicating the changes in response to a 6 h induction by different concentrations of H_2_O_2_ in NOR and MCD keratocytes (**k**). Error bars indicate SD.

Pyroptosis can protect cells, but it may also contribute to cell death^34^. Exposure of normal and MCD keratocytes to different concentrations (0–200 μM) of the reactive oxygen species (ROS) H_2_O_2_ for 6 h resulted in a dose-dependent increase in levels of the pyroptosis-related proteins NLRP3, caspase-1, Pro-IL-1β, and IL-1β in both normal and MCD keratocytes, but the increases were greater in the MCD keratocytes. At higher H_2_O_2_ concentrations (400–600 μM), the expression levels of NLRP3, caspase-1, Pro-IL-1β, and IL-1β showed distinct decreases, which may have reflected increased cell death caused by high H_2_O_2_ concentrations (Fig. 4a–e, f–j). Figure 4k shows that the *CHST6* mutation increased the susceptibility to H_2_O_2_-induced pyroptosis in MCD. All these results suggested activation of ROS–NLRP3 inflammasome-induced pyroptosis and an inflammatory pathway in cultured MCD keratocytes and further aggravation by H_2_O_2_ stimulation^35^.

### Caspase-1 inhibitor, Ac-YVAD-CMK, could attenuate the development of H_2_O_2_-induced pyroptosis in normal and MCD keratocytes

**Fig. 5:**
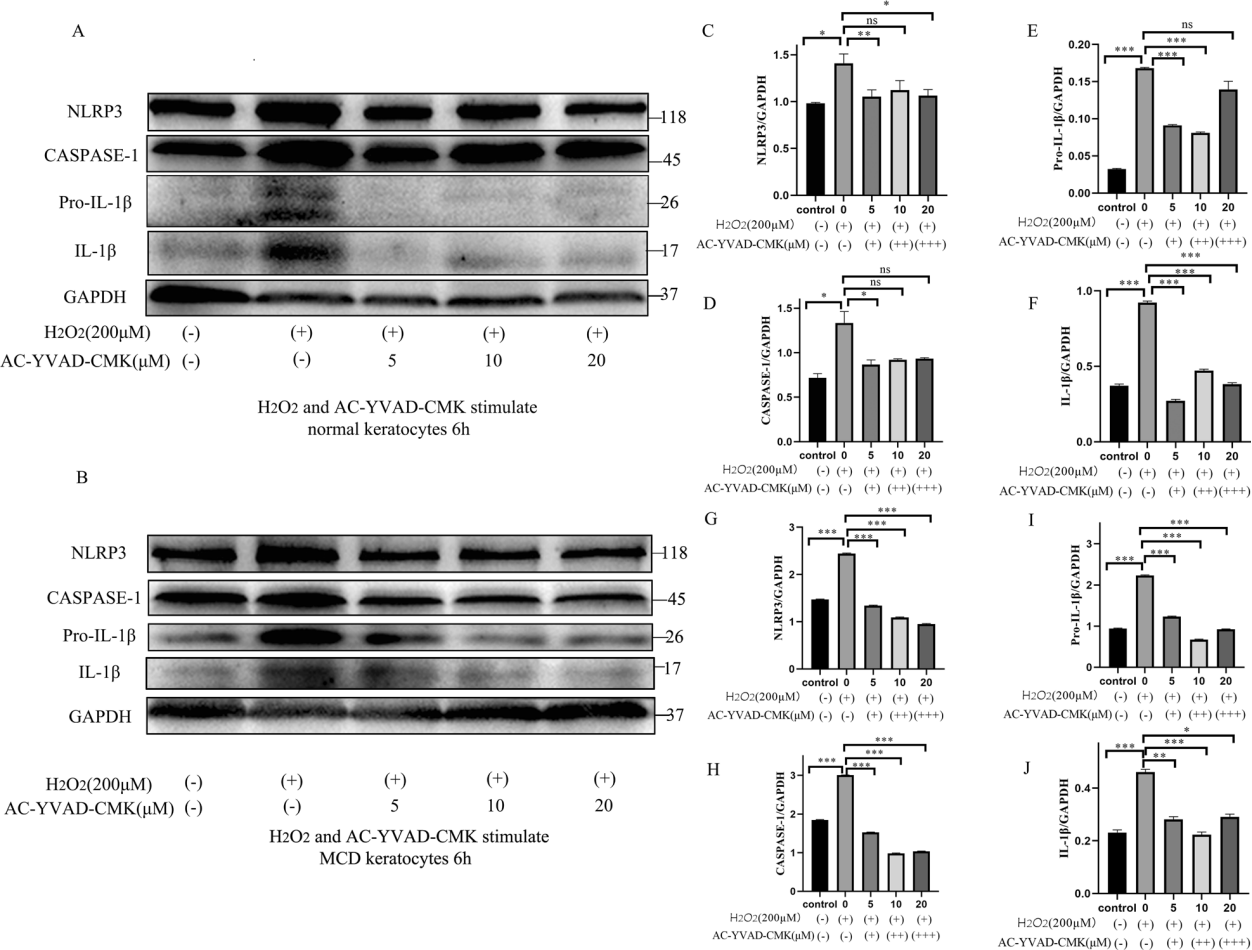
The changes in the levels of pyroptosis in NOR and MCD keratocytes induced by 6 h induction with 200 μM H_2_O_2_ in the presence of different concentrations of the caspase-1 inhibitor Ac-YVAD-CMK. Caspase-1 antagonism and the changes in the production of pyroptosis-related proteins were determined by western blotting. Ac-YVAD-CMK decreased the expression of H_2_O_2_-induced pyroptosis-related proteins (NLRP3, caspase-1, Pro-IL-1β, and IL-1β) in normal keratocytes. The expression of H_2_O_2_-induced pyroptosis-related proteins in the normal keratocytes was reversed by Ac-YVAD-CMK (**a**). *p < 0.05, **p < 0.01, ***p < 0.001, ns = no significance versus the untreated group (200 μM H_2_O_2_ and 0 μM Ac-YVAD-CMK); *n* = 3 for each group; One-way ANOVA (**c**–**f**). Ac-YVAD-CMK reduced the expression of H_2_O_2_-induced pyroptosis-related proteins (NLRP3, caspase-1, Pro-IL-1β, and IL-1β) in MCD keratocytes. The expression of H_2_O_2_-induced pyroptosis-related proteins in MCD keratocytes was prevented by treatment with Ac-YVAD-CMK (**b**). *p < 0.05, **p < 0.01, ***p < 0.001 versus the untreated group (200 μM H_2_O_2_ and 0 μM Ac-YVAD-CMK); *n* = 3 for each group; One-way ANOVA (**g**–**j**). Error bars indicate SD.

The caspase-1 inhibitor Ac-YVAD-CMK structurally resembles the catalytic substrate of caspase-1 and irreversibly antagonizes caspase-1 activity^36^. Ac-YVAD-CMK protects against H_2_O_2_-aggravated pyroptosis injury in normal and MCD keratocytes by suppressing caspase-1-mediated pyroptotic cell death through downregulation of the protein expression of NLRP3 and caspase-1, as well as hampering Pro-IL-1β which was cleaved into mature IL-1β. Measurement of the expression of the NLRP3 inflammasome signaling pathway proteins (Fig. 5a, b) revealed that H_2_O_2_ induction significantly augmented the expression of pyroptosis-related proteins in the untreated group (200 μM H_2_O_2_ and 0 μM Ac-YVAD-CMK), compared with the control (0 μM H_2_O_2_ and 0 μM Ac-YVAD-CMK) normal and MCD keratocytes. However, Ac-YVAD-CMK treatment inhibited the NLRP3 inflammasome activation by downregulating the protein levels of NLRP3, caspase-1, Pro-IL-1β, and IL-1β in normal and MCD keratocytes co-treated with H_2_O_2_ (200 μM) and with various doses of Ac-YVAD-CMK when compared with the untreated group (200 μM H_2_O_2_ and 0 μM Ac-YVAD-CMK) (Fig. 5a, b). The expression of NLRP3, caspase-1, Pro-IL-1β, and IL-1β protein was also dramatically downregulated by the inhibitor in normal and MCD keratocytes (Fig. 5c–j). The optimal concentration of Ac-YVAD-CMK appeared to be 5 and 10 μM for suppression of pyroptosis in normal and MCD keratocytes induced by 200 μM H_2_O_2_ (Fig. 5a–j). Caspase-1 inhibition reversed H_2_O_2_-aggravated pyroptosis by decreasing caspase-1 activity and the production of precursor and mature IL-1β in normal and MCD keratocytes, implying that the protective effects of Ac-YVAD-CMK against H_2_O_2_-aggravated pyroptosis were mediated by its effects on caspase-1-dependent pyroptosis activation.

### H_2_O_2_ treatment revealed a relationship between sulfated KS and pyroptosis in MCD keratocytes

**Fig. 6:**
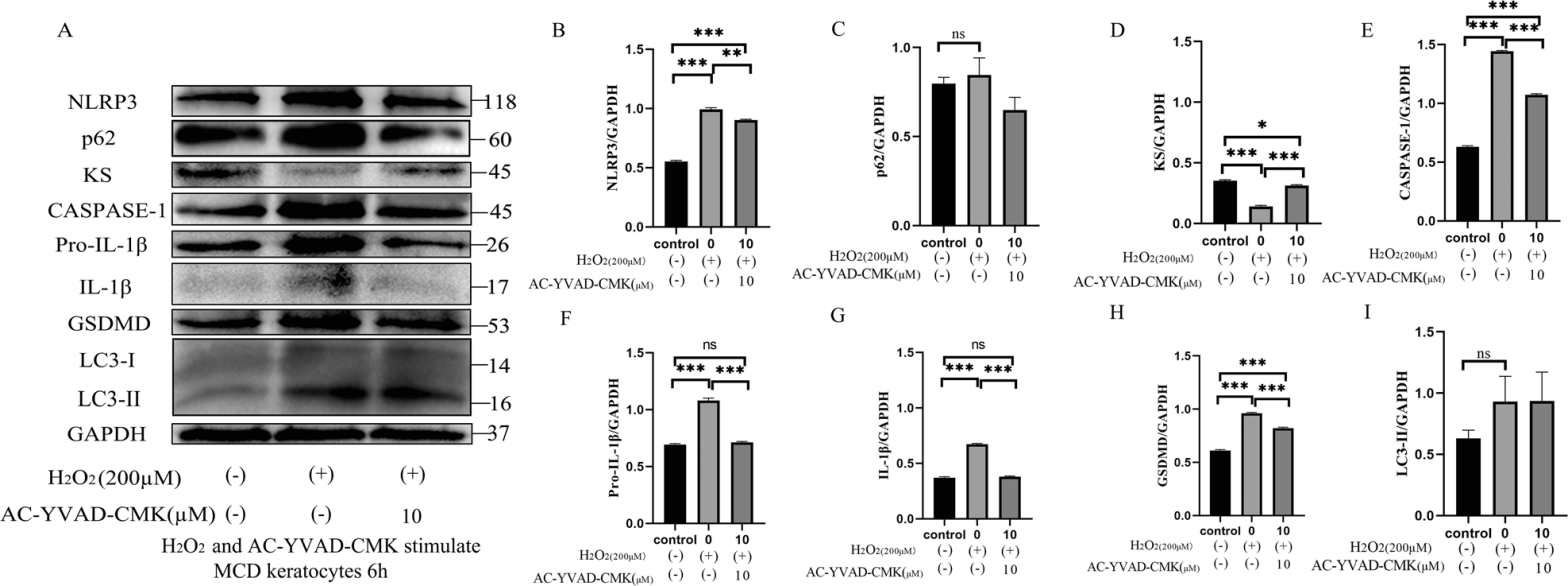
Autophagy, pyroptosis, and production of sulfated KS in MCD keratocytes. Western blot analyses of the expression of H_2_O_2_-induced (200 µM) pyroptosis-related proteins, sulfated KS in the presence and absence of Ac-YVAD-CMK (10 μM), and the expression of p62 and LC3-II in MCD keratocytes (**a**). *p < 0.05, **p < 0.01, ***p < 0.001, ns = no significance versus the control (0 μM H_2_O_2_ and 0 μM Ac-YVAD-CMK) and untreated (200 μM H_2_O_2_ and 0 μM Ac-YVAD-CMK) group; *n* = 4 for each group; One-way ANOVA (**b**–**i**). Error bars indicate SD.

Exogenous H_2_O_2_ induces autophagy, as demonstrated in many reports^37–40^, and our data indicated that H_2_O_2_ can induce pyroptosis. The expression of p62 and LC3-II proteins in H_2_O_2_-induced (200 µM) MCD keratocytes changed meaninglessly from the levels in untreated (200 μM H_2_O_2_ and 0 μM Ac-YVAD-CMK) and control (0 μM H_2_O_2_ and 0 μM Ac-YVAD-CMK) group. However, pyroptosis-related proteins were significantly upregulated in H_2_O_2_-induced (200 µM) MCD keratocytes compared with the control (0 μM H_2_O_2_ and 0 μM Ac-YVAD-CMK) group (Fig. 6a–c, e–i), confirming that H_2_O_2_ (200 µM) had no pronounced effects on autophagy in MCD keratocytes. These results also showed that Ac-YVAD-CMK (10 µM) can dramatically mitigate a H_2_O_2_-aggravated pyroptosis insult by decreasing NLRP3 inflammasome activation, thereby reducing the pyroptosis induced by activated caspase-1 and the inflammatory response in MCD keratocytes (Fig. 6a, b, e–h).

Normal antigenic KS can also be detected in MCD keratocytes. Interestingly, the promotion of cell pyroptosis significantly reduced the expression of sulfated KS and the inhibition of cell pyroptosis distinctly augmented the expression of sulfated KS in MCD keratocytes (Fig. 6a, d). Previous evidence indicated that several small leucine-rich proteoglycans which were glycosylated with KS ultimately formed the KS proteoglycan (KSPG) and sustained the correct interfibrillar spacing of the collagen fibrils in the corneal tissue^41^. Non-sulfated KS has been discovered in MCD keratocytes, and it is produced in equivalent amounts to normal KS in the normal cornea. Antibodies to the core protein of normal KSPG react with the non-sulfated KSPG found in MCD I^42^. Thus, the non-sulfated KSPG may be expelled from the MCD keratocytes into the corneal stromal layer via cell pyroptosis, further aggravating the symptoms of MCD.

## Discussion

The expression of p62 and LC3-II proteins was upregulated in MCD keratocytes compared with normal keratocytes, suggesting that autophagy was triggered and autophagic flux was impaired in MCD keratocytes. We suspected that MCD might cause disturbances in the autophagic degradative system as a result of autophagosome-lysosome fusion or lysosomal dysfunction. The autophagy inhibitor bafilomycin-A1 increased p62 and LC3-II protein levels in normal and MCD keratocytes, accompanied by a decrease in the LysoTracker signal and CTSD expression in MCD keratocytes. These findings supported the possibility that autophagy defects are likely due to lysosomal dysfunction in MCD.

Corneal glucosamine C-GlcNAc6ST is encoded by *CHST6* on chromosome 16q22. This enzyme transfers sulfate to the unsulfated keratan chains, utilizing 3’-phospho-5’-adenylyl sulfate as a sulfonate donor to catalyze the transfer of sulfate to position 6-O of the N-acetyl-glucosamine of keratan in the cornea^6,43^. Mutations in C-GlcNAc6ST, such as in the 5′PB domain that contains an essential part of the active site responsible for 3′-phospho-5′-adenylyl sulfate binding, seriously impact sulfotransferase activity and result in the deposition of poorly sulfated and non-sulfated KS in the intracellular and extracellular matrix of the MCD cornea^44^. In its non-sulfated form, KS was translocated via its signal peptide into the ER, where it then matured during passage through the Golgi. Non-sulfated KS may be secreted via Golgi-derived secretory vesicles into the cytoplasm, where it is ultimately deposited due to a lack of hydrophilicity. Accumulation of non-sulfated KS then persists due to impaired autophagy, and non-sulfated KS is eventually stored in the lysosomes. The non-sulfated KS that accumulates in MCD lysosomes might be secreted into the ECM via an unconventional autophagy-based secretory route recently reported for the extracellular delivery of IL-1β^45^. In the present study, H_2_O_2_-induced pyroptosis decreased the level of sulfated KS, while Ac-YVAD-CMK inhibition of pyroptosis restored the expression of sulfated KS in MCD keratocytes, probably by cuing non-sulfated KS excretion into the ECM via pyroptosis. Our previous research showed that expression of the ER stress marker protein GRP78 and CHOP protein were enhanced in MCD keratocytes and triggered the ER stress response and CHOP-mediated apoptosis^22^. ER-associated degradation is tightly associated with the activation of ER stress and serves to eliminate misfolded proteins from the ER via the cytoplasmic ubiquitin-proteasome system^46^. Therefore, the non-sulfated KS that accumulates in the ER might also be degraded by the cytoplasmic ubiquitin-proteasome system. Aggregates of non-sulfated KS may undergo exocytosis mediated by Golgi-derived secretory vesicles and exosome pathways via plasma membrane fusion and excretion into ECM^47^. These pathways for intracellular metabolism and secretion into ECM suggest the possibility that aggregates of non-sulfated KS might also be secreted by pyroptosis and ultimately be deposited in the corneal matrix to cause cornea opacity.

The ocular surface inflammatory status has been investigated in different types of CD^48^, but the potential molecules involved in the pathogenesis of the inflammatory response in MCD keratocytes remains underexplored. TEM and histochemical observations confirmed the presence of aggregations in the vacuolated cytoplasm, the occurrence of cell membrane rupture, and the accumulation of granular material in MCD keratocytes (Supplementary Fig. 1). The pyroptosis-related proteins NLRP3, caspase-1, Pro-IL-1β, IL-1β, and GSDMD were upregulated in MCD keratocytes, supporting an involvement of pyroptosis in MCD. Notably, the keratocytes were characterized by intracytoplasmic aggregations of hydrophobic, non-sulfated KS, which may be perceived as a DAMP, resulting in inflammatory processes that ultimately lead to disease progression. Our findings highlighted that canonical inflammasome-induced pyroptosis may play a major role in the inflammatory response of MCD. Canonical inflammasome-induced pyroptosis involves immune activators, such as DAMPs, pathogen-related molecular patterns, other exogenous invaders, or environmental stress, that activate the interaction between the pyrin domains in NLRP3 and apoptosis-associated speck-like protein. The caspase recruitment domain of apoptosis-associated speck-like protein recruits procaspase-1 to trigger caspase-1 generation of GSDMD-N, mature IL-1β, and IL-18. This promotes cell membrane rupture and a pronounced inflammatory response^49^. By contrast, the non-canonical pathway appears to involve the discernment of cytosolic lipopolysaccharide (LPS) from invading gram-negative bacteria by caspases (murine caspase-11 and human homologs caspase-4 and 5), followed by direct cleavage of GSDMD by caspase-4/5/11 directly into a C-terminal fragment and an N-terminal fragment. The N-terminal domain of GSDMD forms extensive gasdermin pores and provokes caspase-1 to cleave pro-IL-1β/18 in accordance with the canonical inflammasome-induced pyroptosis^50^. Previous work has shown that antigenic KS was absent from the corneas and blood sera of patients with MCD type I and was solely present in the keratocytes^23^. In MCD type II, normal antigenic KS was detected in both the cornea and serum but at lower levels than seen in normal keratocytes^24,25^. These findings hint that pyroptosis may be involved in a phenotype of MCD that is potentially attributed to the cell membrane rupture causing the release of KS protein into the corneal stroma layer.

H_2_O_2_ treatment can promote the assembly and activation of the NLRP3 inflammasome, triggering caspase-1 to initiate the production of IL-1β, as well as IL-18^35^. H_2_O_2_ is also pivotal for autophagosome formation and autophagic degradation, as it mediates multiple signaling pathways. The oxidative signal is partially PI3K dependent and contributes to inhibition of Atg4, ultimately facilitating autophagy^51^. ROS accumulation suppresses PI3K/AKT/mTOR signaling and sequentially augments autophagy^52^. Our data demonstrated that the expression levels of p62, LC3-II, and pyroptosis-related proteins were increased in MCD keratocytes treated with H_2_O_2_ (200 µM). Therefore, H_2_O_2_-induced (200 µM) pyroptosis was superior to induce autophagy in MCD keratocytes. However, we cannot rule out the possibility that the level of H_2_O_2_ used (200 µM) was not sufficient for autophagy induction in our system. Administration of Ac-YVAD-CMK substantially diminished the protein-level expression of NLRP3, caspase-1, pro-IL-1β, IL-1β, and GSDMD in H_2_O_2_-induced MCD keratocytes. The treatment with Ac-YVAD-CMK inhibited H_2_O_2_-induced pyroptosis in MCD keratocytes apparently by restraining the expression of caspase-1 and production of pivotal downstream inflammatory cytokines (IL-1β and IL-18), as well as by hampering GSDMD cleavage. These results point to a vital therapeutic potential of caspase-1 inhibition that deserves clinical consideration. Notably, local application of Ac-YVAD-CMK in the form of eye drops might be proved effective in decreasing the corneal opacification associated with MCD.

**Fig. 7:**
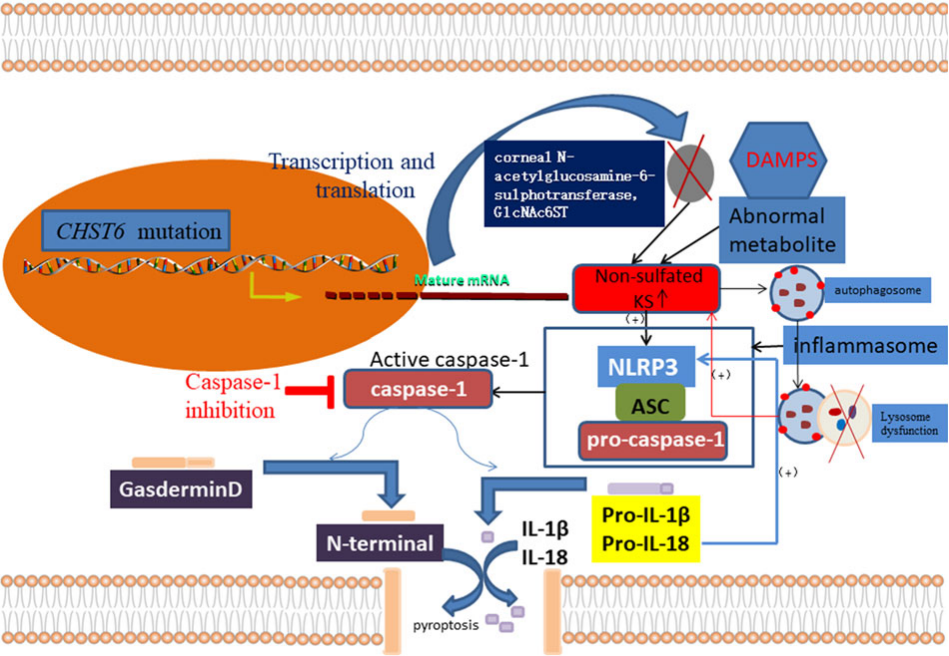
A graphical model of the pyroptosis effects of various molecules on MCD keratocytes. *CHST6* gene mutation and impairment of the autophagy signaling pathway induce excessive production of non-sulfated KS. Non-sulfated KS aggregations form clinically phenotypic features, depositing in the corneal stromal extracellular matrix and causing focal patchy white opacity. A chronic overload of non-sulfated KS aggregations, as a DAMP, triggers the NLRP3 inflammasome and keratocytes undergo pyroptosis via the intrinsic pathway. The caspase-1-mediated pyroptosis signaling pathway might be a latent therapeutic target for treatment of MCD.

In summary, a family with a homozygous *CHST6* mutation was identified in Northeast China (Supplementary Fig. 2). We propose the possibility that impaired autophagy activates the NLRP3-caspase-1 inflammasome pathway^53^ and the subsequent activation of apoptosis triggers pyroptosis via a caspase-3-mediated pathway that results in cleavage of GSDME^54,55^. Multiple signaling pathways participate in MCD and determine cell fate. We postulate that pyroptosis is implicated in the pathogenic processes of MCD (Fig. 7). Taken together, our findings indicate that supplementation with the CTSD enzyme and administration of Ac-YVAD-CMK may represent a therapeutic strategy for the prevention of MCD.

## Materials and methods

### Patients and control subjects

Three patients from 3 unrelated families received clinical diagnoses of MCD by three doctors using slit-lamp biomicroscopy in the First Affiliated Hospital of Harbin Medical University; anterior segment optical coherence tomography was also analyzed in this study. Control subjects were selected from 50 individuals with no visual impairment. Informed consent was obtained from all patients and their family members in agreement with the Declaration of Helsinki for research involving human subjects. Approvals for genetic testing were obtained from the First Affiliated Hospital of Harbin Medical University.

### Materials

The following Reagents and antibodies were used in this study: Dulbecco’s Modified Eagle Media: Nutrient Mixture F-12 (Hyclone, UT, USA); 1 × phosphate buffered saline (1 × PBS; Hyclone, UT, USA); Fetal bovine serum (ExCell Bio, Australian); Hydrogen peroxide (H_2_O_2_) (Chemistry Industry Co, Shandong, China); Cell Counting Kit-8 (Beyotime, Shanghai, China); ECL developer (Beyotime, Shanghai, China); caspase-1 inhibitor Ac-TYR-VAL-ALA-ASP-CMK (Ac-YVAD-CMK) (Cayman Chemical, MI, USA); lysosomal inhibitor bafilomycin-A1 (Aladdin, Shanghai, China); Primary antibodies: anti-NLRP3, anti-caspase-1, anti-IL-1β, anti-CTSD, and anti-GSDMD (Abcam, Cambridge, MA, USA); anti-keratan sulfate (5D4) (MD bioproducts, MN, USA); anti-GAPDH (Proteintect, IL, USA); anti-p62 and anti-LC3 (Cell Signaling Technology, MA, USA); TWEEN-20 (Solarbio, Beijing, China); HRP-conjugated secondary antibody (Zsbio, Beijing, China); LysoTracker Green DND-26 (Cell Signaling Technology, MA, USA).

### Methods

#### Limbus tissue primary culture and cell treatment

Primary keratocytes were acquired from normal donors from the Heilongjiang Province Eye Bank and from mutation-type MCD patients who underwent penetrating or lamellar keratoplasty at the First Affiliated Hospital of Harbin Medical University. Cells were represented by 3 unrelated families and various MCD patients and 3 various normal donors. The limbus tissue of the MCD patients in this study and the limbus tissue of normal donors were taken for primary culture. The tissues were washed three times with 1 × PBS containing 500 U/ml penicillin and 500 µg/ml streptomycin, and the corneal epithelium, endothelium, sclera, and conjunctiva were removed completely. The limbus tissue was then carefully separated and disintegrated into 1–2 mm^3^ pieces, and complete culture media was added after achieving full attachment of the tissues. The primary cell cultures were incubated at 37 °C in 5% CO_2_. The primary cell cultures were observed daily, and the medium was changed every 3 or 4 days. For subculturing, the cells were digested with 0.05% trypsin and 5.0 mmol/L Ethylene Diamine Tetraacetie Acid (Gibco, NY, USA) when the density of the cells had reached 80–90% and the cells were transferred to culture flasks containing complete culture media.

#### DNA extraction and gene sequencing

Genomic DNA was isolated from peripheral blood leukocytes of MCD patients using a DNA extraction kit (Tiangen, Guangzhou, China) according to the manufacturer’s instructions. Three pairs of primers (1, 2, and 3, designed by Akama et al.) were used to amplify the region of the open reading frame of *CHST6* (ref. ^6^). Each PCR reaction was performed in a 50 μL reaction mixture consisting of genomic DNA (100 ng) and 25 μL of PrimeSTAR HS Premix DNA polymerase (Takara, Kusatsu, Japan) containing PrimeSTAR HS DNA Polymerase, dNTP Mixture and PrimeSTAR HS Buffer. Amplification reactions were performed under the following conditions: 5 min of denaturation at 98 °C, followed by 35 cycles of denaturation at 98 °C for 10 s, annealing for 15 s at 55 °C (for the middle coding region) or at 57 °C (for the 5′ and 3′ coding regions), extension at 72 °C for 45 s, and a further extension step at 72 °C for 7 min. The PCR products were separated by agarose gel electrophoresis. Bidirectional sequencing was performed with a DNA sequencer (model 3730; Applied Biosystems, CA, USA).

#### Treatment of keratocytes

Cells in the logarithmic growth phase were collected and co-cultured with various concentrations of H_2_O_2_ and Ac-YVAD-CMK for the required time periods. H_2_O_2_ was diluted in Dulbecco’s Modified Eagle Media: Nutrient Mixture F-12 and used at a 10 mM concentration. Ac-YVAD-CMK and bafilomycin-A1 were dissolved in dimethyl sulfoxide, and used at 20 mg/mL and 1 mg/mL, respectively, and then diluted in Dulbecco’s Modified Eagle Media: Nutrient Mixture F-12.

#### Inverted phase-contrast microscopy

Cell morphology was observed by inverted phase-contrast microscopy when cells had entered the logarithmic growth phase. After treatment, the cell morphology was observed with an inverted phase-contrast microscope, and digital images were captured.

#### Histological and transmission electron microscopy (TEM) observation of corneal tissue and Keratocytes

Histological and TEM studies were performed on corneal flaps obtained from patients with MCD who had undergone corneal transplantation. Histological analysis of 4 μm thick sections of paraffin-embedded corneal flap was performed in a patient with MCD after keratoplasty. The corneal tissues were stained with periodic acid Schiff stain and Alcian blue for light microscopy. Keratocytes and corneal tissues were also prepared for thin sections by fixing specimens with 2.5% (v/v) glutaraldehyde in 0.1 M for 2 h, rinsing with 3 changes of 1 × PBS; and post-fixing with 1% (v/v) OsO_4_ in 1 × PBS for 2 h. The specimens were washed, dehydrated in a graded ethanol series, and embedded in epoxy (low-viscosity agar) resin following standard protocols. Ultrathin sections were collected on carbon-coated 100 mesh copper grids and stained with 1% uranyl acetate and 1% lead citrate. The sections were viewed at 80 kV in a Hitach-H7650 TEM.

#### Western blotting

Cells were collected and lysed in RIPA buffer (Beyotime, Shanghai, China) containing 1% phenyl methyl sulfonyl fluoride (Beyotime, Shanghai, China). The protein concentration was calculated with a bicinchoninic acid protein assay kit (Beyotime, Shanghai, China). The protein was separated on a 12.5% sodium dodecyl sulfate-polyacrylamide gel electrophoresis (SDS-PAGE) gel and then transferred to a polyvinylidene difluoride (0.22 μm) membrane (Millipore Corp, Atlanta, GA, USA) by wet transfer. After blocking with 8% skimmed milk powder, the polyvinylidene difluoride membrane was incubated overnight with the following main antibodies at 4 °C: anti-NLRP3, anti-caspase-1, anti-IL-1β, anti-GSDMD, anti-p62, anti-LC3, and anti-CTSD were respectively at 1/1000 dilution. Anti-keratan sulfate (5D4) was at 1/250 dilution and anti-GAPDH was at 1/2000 dilution. After three washes in PBST (1 × PBS containing 0.05% TWEEN-20), the membrane was incubated with the appropriate HRP-conjugated secondary antibody (1:5000) for 1 h at room temperature using ECL developer. An imaging system (Tanon 4600, Shanghai, China) was used to detect and analyze the signals.

#### Real-time quantitative reverse transcription PCR

Total RNA was extracted from cells using Trizol reagent (Invitrogen, CA, USA) according to the manufacturer’s instructions, the concentration of RNA was detected by a NanoDrop Spectrophotometer (NanoDrop Technologies, Wilmington, DE, USA), and 1 μg of total RNA was reverse transcribed into cDNA using a Reverse Transcription Kit (Takara, Kusatsu, Japan). The Reverse Transcription reaction consisted of 1 μg RNA, 2 μL 5 × RT Buffer, 0.5 μL primer Mix, 0.5 μL RT Enzyme Mix, and 6 μL nuclease-free water in a total volume of 10 μL. Reactions were performed in an Eppendorf PCR System (Eppendorf, Hamburg, Germany) for 15 min at 37 °C, followed by heat inactivation for 5 min at 95 °C. The 10 μL RT reaction mix was then held at −20 °C. Primers for NLRP3, caspase-1, IL-1β, and GAPDH were designed and synthesized by Corporation (Sangon, Shanghai, China). A 480 II Real-time PCR Instrument (Roche, Basel, Switzerland) was used with a 20 μL PCR reaction mixture that included 2 μL cDNA, 10 μL 2 × LightCycler® 480 SYBR Green Realtime PCR Master Mix (TOYOBO, Osaka, Japan), 0.8 μL forward primer, 0.8 μL reverse primer, and 6.4 μL nuclease-free water. Reactions were incubated in a 96-well optical plate at 95 °C for 30 s; followed by 40 cycles of 95 °C for 5 s, 55 °C for 10 s, and 72 °C for 15 s. Each sample was run in triplicate for analysis. At the end of the PCR cycles, melting curve analysis was performed to validate the specific generation of the expected PCR product. All experiments were done in triplicate. The expression levels of RNAs were normalized to glyceraldehyde-3-phosphate dehydrogenase and were calculated using the 2^−ΔΔCt^ method.

#### Cell proliferation assay

A cell proliferation test kit was used following the manufacturer’s instructions. Keratocytes from patients with MCD and a healthy control group were inoculated into 96-well plates, and a Cell Counting Kit-8 solution was added after appropriate time (24 h, 48 h, and 72 h). Before detection, 10 μL of CCK-8 reagent was added to the culture medium in each well and the plate was incubated in 37 °C in 5% CO_2_ incubator for 2 h. The absorbance at 450 nm was read with a microplate reader (Bio-Rad, CA, USA) and all experiments were performed in triplicate.

#### LysoTracker Green DND-26 labeling and FACS analysis

LysoTracker Green dye stains cellular acidic compartments and visualizes enlarged lysosomes. Keratocytes were stained with LysoTracker Green according to the manufacturer’s instructions by incubating them with the dye for 30 min at 37 °C. The proportion of LysoTracker-positive cells was measured by flow cytometry.

#### Statistical methods

The measurements were expressed as means ± standard deviation. One-way ANOVA was used to compare the data of multiple groups, and a Student’s *t*-test was used to compare the data of two groups. All statistical analyses were performed using GraphPad software (GraphPad Prism 8, GraphPad Software, La Jolla, CA, USA). A value of *p* < 0.05 was considered significant (*p < 0.05, **p < 0.01, ***p < 0.001, ns = no significance). The individual experiments were repeated at least three times and representative data are displayed.

## Supplementary information

supplementary figure legends

supplementary Figure 1

supplementary Figure 2

supplementary Figure 3
