# Supplementary material for: Impairment of the autophagy-lysosomal pathway and activation of pyroptosis in macular corneal dystrophy
Source: Cell Death Discov. 2020 Sep 12;6:85. doi: 10.1038/s41420-020-00320-z (PMC7487068; doi:10.1038/s41420-020-00320-z)
Supplement: Supplementary file 1 — supplementary figure legends [file 41420_2020_320_MOESM1_ESM.docx]

**Supplementary Figure 1:** TEM appearance (A-D). Enlargement of intracytoplasmic vacuoles and the presence of intracellular electron-dense granules were observed in MCD keratocytes compared with NOR, which had intact cell membranes, as indicated by the arrow. Histopathological appearance (E-J) showed small and large vacuoles containing acid mucopolysaccharide material that stained positively with periodic acid Schiff and Alcian-blue stains, as indicated by the arrow.

**Supplementary Figure 2:** Clinical and genetic aspects: (A, D) One novel homozygous frameshift mutation in family 9, c.290-291 insG. (B) Ophthalmic slit lamp examination showed diffuse haze turbidity and focal patchy white opacity in both eyes of the progressive corneal stroma. (C) Genetic family diagram shows MCD is an autosomal recessive genetic disease.

**Supplementary Figure 3:** Without any intervention, the expression of caspase-1 and cleavage caspase-1 p20 was detected in NOR and MCD keratocytes by western blotting: (A-C) Western blotting revealed significantly higher levels of caspase-1 and cleavage caspase-1 p20 in MCD keratocytes than in NOR keratocytes. *p<0.05 versus normal keratocytes; Student’s t test; n = 3 derived from each of three lines. Error bars indicate SD.
