# Supplementary figures and images for: Impairment of the autophagy-lysosomal pathway and activation of pyroptosis in macular corneal dystrophy
Source: Cell Death Discov. 2020 Sep 12;6:85. doi: 10.1038/s41420-020-00320-z (PMC7487068; doi:10.1038/s41420-020-00320-z)

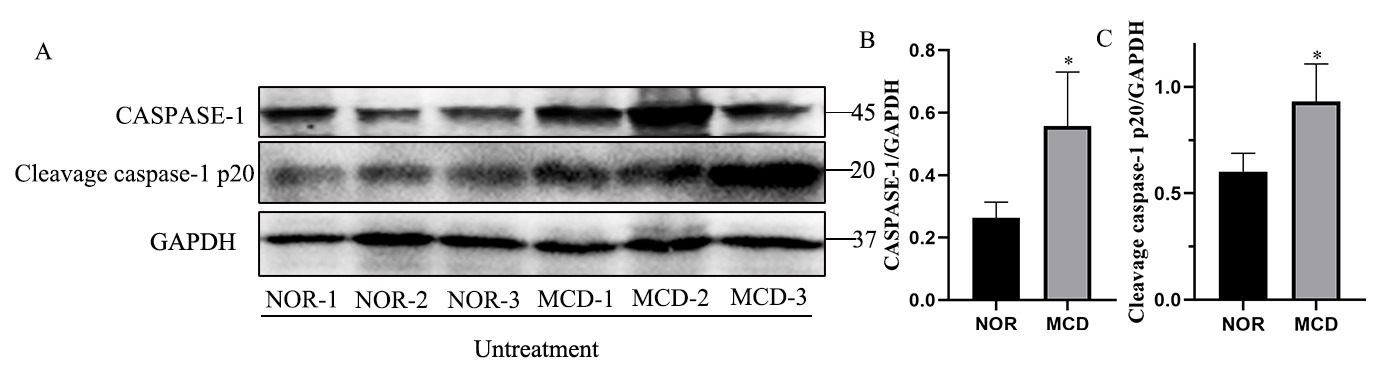

Supplement: Supplementary file 4 — supplementary Figure 3 [file 41420_2020_320_MOESM4_ESM.png]
